# Can vertical environmental regulation become a sharp weapon in China's green development process? The moderating role of pollution dividend

**DOI:** 10.3389/fpubh.2023.1113457

**Published:** 2023-02-15

**Authors:** Sixuan Du, Haiying Pan, Zuhan Meng, Ni Wang, Jiajia Ren

**Affiliations:** ^1^Business School, Hohai University, Nanjing, China; ^2^Jiangsu Research Base of Yangtze Institute for Conservation and High-Quality Development, Nanjing, China; ^3^Yangtze Institute for Conservation and Development, Nanjing, China

**Keywords:** vertical environmental regulation, green development efficiency, pollution dividend, spatial Durbin model, moderating effect

## Abstract

Optimizing the vertical environmental regulation (VER) effect of the central government and reducing the negative execution motivation of local governments have become the priority points to accelerate the green development of China. Based on the spatial Durbin model, this paper not only examines the influence of VER on green development efficiency (GDE), but also discusses the moderating effect of politically and economically motivated pollution dividend (PPD and EPD) on the relationship between them. The research results are as follows: (1) VER has a U-shaped effect on local GDE, the green governance effect of which began to appear when VER was higher than 1.561. VER has an inverted N-shaped effect on adjacent GDE. When the VER intensity lies in (0.138, 3.012), it has a positive spatial spillover effect. (2) PPD weakens the local green governance effect of VER, while EPD positively moderates it. Both of them have no significant moderating effect on it in neighboring areas. (3) Cross-regional cooperative governance moderates the short-term weakness and pollution transfer of VER, and generally facilitates the positive moderating effect of PPD and EPD. In China's two major economic belts, VER, PPD and EPD also have different performances. This study proves the important influence of local inter-governmental competition and promotion tournament on the central environmental regulation for the first time, which is of great significance for optimizing the top-level design of the central government and implementing the governance responsibility of local governments.

## 1. Introduction

**Figure 1:**
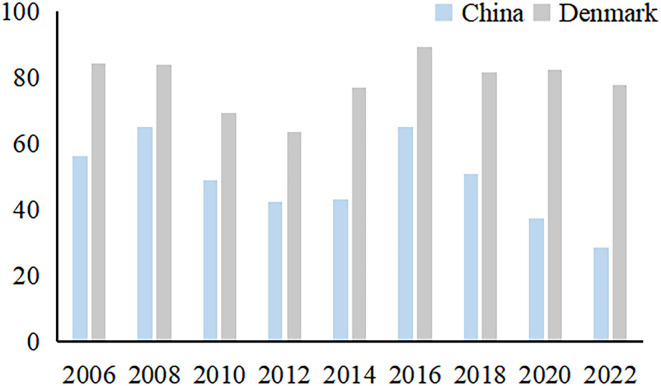
Comparison of EPI between China and Denmark.

Currently, resource mismatch, environmental pollution, and ecological degradation have become prominent bottlenecks limiting China's sustainable economic development. According to the Global Environmental Performance Index (EPI) report released by Yale University and other institutions, China's EPI tends to decline in fluctuation from 2006 to 2022, even as low as 28.4 in 2022, with a gap of 49.5 with Denmark, which ranks first in the world (see Figure 1), reflecting that China's environmental performance has much room for improvement. At the same time, over the last decade or so, China has been committed to promoting a shift from the traditional crude development mode to the green development mode. In particular, since the 18th Party Congress, the central government have adhered to the law enforcement concept of “fight pollution with an iron hand”, and enacted the strictest environmental regulations in many ways to ensure that “green” becomes the color of China's economic development.

We have noticed that under the decentralized governance system of central and local governments, China has implemented dual environmental regulations, that is, vertical environmental regulation (VER) and parallel environmental regulation go hand in hand. Among them, VER promotes polluting enterprises to complete green transformation as soon as possible by strengthening central monitoring and punishment, which is more authoritative than parallel environmental regulation in theory. So, in practice, does VER achieve the expected green governance effect? Is the green governance effect affected by other factors? The investigation of the above problems is of great significance for China to find the focus of environmental policy and promote the green development process under the double-cycle pattern.

In academic circles, the research on green governance effect of environmental regulation has become a hot topic in recent years. Some scholars have discussed the optimal regulatory intensity of environmental regulation, such as the rationality verification and expansion research of Porter's hypothesis in the field of green development (1, 2); Some scholars have investigated the optimal structure of environmental regulation, such as distinguishing environmental regulation with government, market and public as the main body and making a comparative analysis of green governance effect (3, 4); Other scholars have further explored whether local government competition (5), foreign direct investment (6) and financial development (7) have optimized or weakened the green governance effect of environmental regulation. However, we find that the research on the governance effect of VER is still lacking. According to the existing research, when dealing with external problems such as pollution, if it can be clearly assessed, the effect of VER will be better than that of territorial management (8). It is the core of China's future environmental decentralization reform to implement comprehensive VER to supplement parallel environmental regulation (9). Therefore, this paper tries to test whether the green governance effect of VER can live up to expectations in the process of China's green development.

Under the special decentralization system in China, the self-interest motivation of local governments may affect the green governance effect of VER. We explain this possible regulatory dilemma from a new perspective—pollution dividend (PD). PD, that is, the comparative advantage of environmental factors of production, has evolved from the “theory of environmental factors of production”, meaning that pollution factors can bring economic benefits (10, 11). On the one hand, local governments have sufficient discretion to serve their own interests (12). Under the weak incentive of environmental protection, the resources of the jurisdiction are more used in economic competition and political promotion (13), and it is very common for local governments to distort the will of the central government (14). On the other hand, the central government lacks the real resources to supervise the pollution control of local governments (15). The inefficient central-local feedback mechanism further strengthens the motivation of local governments to “profit” from pollution. The existing literature has discussed the consequences of local governments seeking PD, such as environmental governance behavior floating on the surface (16, 17), selective implementation of environmental policies (18), and difficulty in effective cooperation among local governments (19), etc., but the analysis is often superficial, rarely from the perspective of PD of different motives to explore how local governments influence the green governance effect of VER.

To sum up, in order to reasonably evaluate the green governance effect of environmental regulation, based on decentralization theory, Porter's hypothesis and principal-agent theory, this paper first takes VER led by the central government as the research object, and considers its action mechanism and spatial effect on green development efficiency (GDE). Secondly, politically motivated pollution dividend (PPD) and economically motivated pollution dividend (EPD) of local governments are introduced as moderating variables to explain whether the self-interest motivation of local governments has an impact on the green governance effect of VER under the background of decentralization. Finally, based on different time periods and regions, the heterogeneity of green governance effect of VER is analyzed.

The marginal contribution of this paper is mainly reflected in the following aspects: First, the contribution of the research perspective. Based on the special decentralized governance system in China, this paper discusses the concrete manifestations of VER led by the central government and the self-interest motivation of local governments in the process of green governance. This proves the importance of vertical management in China and the necessity of a “green” promotion system, which provides suggestions for the formation of a collaborative governance pattern of the central and local governments. Second, methodological contribution. Based on the comprehensive goal of “economic growth, environmental optimization, and social benefit improvement”, this paper constructs a more scientific index system of GDE and uses the super-efficiency SBM model with unexpected output to calculate it. This enriches the evaluation dimension of the green development level and lays the foundation for exploring the optimal path of green development in the future. Third, empirical contribution. Based on the spatial Durbin model, this paper comprehensively controls the urban development status of China under the background of green sustainable development through effect decomposition, further tests the effectiveness of regional cooperative governance policy and compares the regional heterogeneity of the two economic belts in China. This provides a China sample for global green development research, which is conducive to formulating more efficient environmental governance policies from the top-level design.

The layout of this paper is as follows: The second part is the theoretical framework and hypothesis; The third part is research design; The fourth part is empirical results and analysis; The fifth part is further discussion; The sixth part is conclusion and prospect.

## 2. Theoretical framework and hypothesis

**Figure 2:**
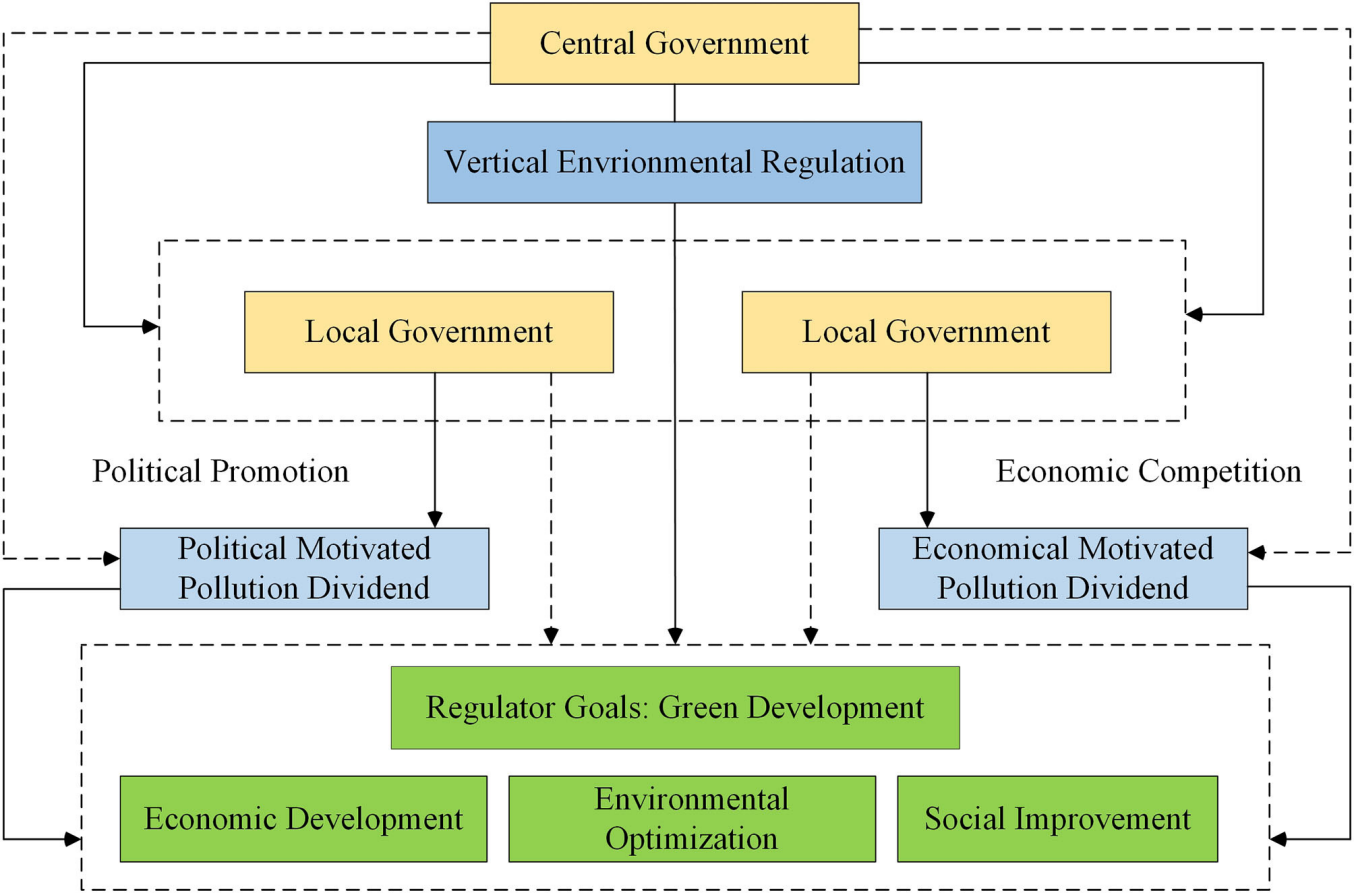
Theoretical framework.

Based on the Chinese-style decentralization background, this paper put VER led by the central government, the local government's pursuit of PPD and EPD, and the green development goal into the same conceptual framework, as shown in Figure 2. This paper follows two clues: First, whether VER can become a sharp weapon in the process of green development; Second, whether the local government's pursuit of PD of different motives has affected the green governance effect of VER. Based on these two clues, this part will review the existing literature and put forward the research hypothesis.

### 2.1. Is vertical environmental regulation effective for green development?

To solve the external diseconomy of pollution, environmental regulation has become the preferred tool for pollution control in all countries without exception (20). In the process of green development, the Chinese government, as the representative of “authoritarian environmentalism”, has sharper tools to promote environmental management than other countries (21, 22). At present, the mechanism of Chinese-style environmental regulation has become the focus of academic circles (23, 24).

Porter's hypothesis points out that reasonable environmental regulation will force enterprises to achieve a win-win situation between economy and environment by upgrading technology (25). However, this is a “beautiful” goal of policymakers. In practice, the “occupation effect” of early environmental protection investment may have a negative impact on the productivity and competitiveness of enterprises (26), which forces polluting enterprises to think about whether to upgrade locally under the “environmental barrier” and “screening effect” or to transfer nearby in the “regulatory difference” and “strategic interaction” (27, 28). The former will gradually get “innovation compensation”, make up for “regulation cost” and provide the engine of long-term green growth for regional development; The latter will not only dampen the local economic vitality, but also lead to the free-riding behavior of transferring the pollution cost to neighboring areas (29, 30), which is not conducive to short-term green development. On the whole, the impact of environmental regulation on local green development may have the characteristics of short-term inhibition and long-term promotion, and may have a negative impact on neighboring green development.

Considering that VER is more authoritative, polluting enterprises that are “specially concerned” by it are more likely to adopt measures such as production reduction, equipment modification, and environmental protection upgrades to cater to the “green” development environment, which will lead to an increase in corporate governance costs and a decrease in product production efficiency in the short term (31). In addition, due to the long transmission chain of VER, the response of polluting enterprises is lagging. Therefore, the green governance effect of VER is difficult to appear in a short time. When enterprises comply with the national strategy and gradually complete the green upgrade, more financial subsidies and tax incentives will bring a broader market for clean products, which not only completes the “de-blackening” of production but also provides basic power for local green growth. Therefore, in the long run, VER will speed up the process of green development. Because of the nonlinear green governance effect of VER, this paper puts forward hypothesis 1:

H1: VER has a U-shaped impact on local green development efficiency.

The difference of regulation will lead to the vicious flight of enterprises. Under the pressure of the central government, some enterprises that can transfer the cost will still move to neighboring areas with lower governance costs at present, in order to avoid the negative effect of VER on the competitiveness of enterprises in the short term, which to some extent increases the difficulty of green development in neighboring areas. Therefore, we believe that VER may have a negative spillover effect in the short term. However, as the difference of regulation becomes smaller, the increase of enterprise transfer costs reduces the pressure of pollution control in neighboring areas. At the same time, in the process of the green governance effect of environmental regulation gradually appearing, the research and development of clean technology, as an important achievement of “innovation compensation” in this region, will also benefit the surrounding areas and drive the green growth of the region. Therefore, we believe that the negative spillover effect of VER will be gradually improved, so this paper puts forward hypothesis 2:

H2: VER has a negative spillover effect on the green development efficiency of neighboring areas, and this negative spillover effect will gradually decrease with the increase of enterprise transfer cost and the research and development of green clean technology.

### 2.2. Does pollution dividend affect the green governance effect of vertical environmental regulation?

China's environmental governance has been undergoing decentralization reform, but when too much power is concentrated in the hands of local governments, certain regulatory deviations will occur. Tahvonen and Kuuluvainen proposed that emission reduction will inevitably require resources, and if more pollution processes are allowed, these resources can be used in other places (10). The theory of environmental factors of production puts forward that environmental pollution is caused by the overuse of environmental factors of production, and this overuse is fundamentally because pollution can bring dividends (11). In recent years, the research on PD is mostly from the perspective of enterprises. On the premise of production technology barriers, China enterprises rely on environmental endowments to mass-produce pollution-intensive products to obtain short-term benefits. This profit strategy of treating pollution as a dividend has become a major reason for China's early extensive growth model (32). Especially, when the cost of pollution factors is low, “rational” enterprises will not choose high technical factors for green transformation, but further choose “laissez-faire” pollution to gain benefits. The “dividend” brought by pollution will make more enterprises flock to it, which will eventually lead to pollution agglomeration (33). However, enterprises' preference for PD is not only due to their lack of environmental awareness but also due to the “acquiescence” of local governments. Yang et al. found in the study of environmental income distribution that the proportion of pollution dividends absorbed by local governments and local enterprises is as high as 60% (32), and even studies have proved that enterprises will capture the strategic regulatory tendency of local governments to purposefully reduce investment in environmental governance (34). Because of the self-interest of the government, local governments are more motivated to pursuit PD than to suppress PD, thus helping them win promotion tournaments and economic competition.

Based on the principal-agent theory, the local government's pursuit of PD may lead to the fluctuation and loss of green governance effect of VER. On the one hand, local governments need to visualize their political achievements to obtain a political promotion, on the other hand, they need to strive for low-cost factors to obtain economic growth. In order to test whether the negative implementation of local governments comes from their role as “political man” or “economic man”, this paper divides the motives for local governments to obtain PD into political motives and economic motives. In order to obtain PPD, local governments tend to take opportunistic pollution control measures. For example, make great efforts to control pollution in the central government's key assessment administrative areas and selectively control pollution in the border areas to prevent the control results from being stolen by neighboring cities (35), or choose to indulge the sewage discharge behavior of enterprises in the jurisdiction after weighing the collusion benefits and potential punishment costs (36), and only adopt extreme environmental regulation to whitewash the pollution control results in the promotion node (37). Therefore, the political promotion intention of local governments has not been completely transformed into the endogenous driving force of green development (38), so we put forward hypothesis 3:

H3: The local government's pursuit of PPD will weaken the green governance effect of VER.

In order to obtain EPD, local governments will fully compete with peer governments to attract liquidity factors, such as competing to relax regulatory standards to reduce the pollution cost of foreign enterprises (39, 40), competing to adjust the fiscal expenditure structure to provide a good investment environment (41). Undoubtedly, technology, capital and high-quality labor force will flow into the jurisdiction one after another due to the excellent regulatory intensity and investment environment, and the agglomeration of these elements will effectively promote the green transformation of the industry in the jurisdiction; At the same time, when the local government does not set an environmental threshold to block foreign investment in highly polluting industries, it may cause pollution factors to gather. Under the pressure of VER, the local government's pursuit of EPD has promoted the gathering of high-quality production factors to a certain extent, and the radiation effect brought by technology and talents will benefit the surrounding areas, which balances the negative effects brought by pollution and is conducive to green development. Therefore, we believe that EPD may promote the green governance effect of VER, so we put forward hypothesis 4:

H4: The local government's pursuit of EPD will optimize the green governance effect of VER.

## 3. Research design

### 3.1. Variable selection

#### 3.1.1. Explained variable: Green development efficiency

Green development is derived from the dual needs of China's structural transformation and ecological civilization construction, and now it has become a hot spot in the economic development of the whole country and even regions (42). Because the conflict faced by the government's environmental regulation decision is the dilemma between economic development and environmental benefits, most of the output indicators of GDE in the existing literature include economic expected output and environmental unexpected output, but rarely include environmental and social expected output (43, 44). Therefore, based on the green concept (45), this paper expands the dimension of expected output to three aspects: economy, environment and society.

In order to reasonably measure the negative impact of environmental pollution, this paper uses a Super Slacks-Based Model (SBM) with unexpected output to measure GDE (46). This model not only corrects the influence of slack variables but also increases the comparability of decision-making units, which is superior to the common DEA model. In the specific selection of input and output indicators, this paper selects capital, labor, technology and resources as input indicators, economic growth, social welfare and pollution control capacity as expected output indicators, and environmental pollution as unexpected output indicators. The specific index system is shown in Table 1, and some indexes are reduced by entropy method.

**Table 1:** Input-output index system of GDE.

**Type**	**Primary index**	**Secondary index**	**Three-level index**
Input	Capital	Fixed-asset investment	Capital stock (47)
	Labor force	Number of employees in the company	Year-end employees
	Technology	Science and technology investment	Fiscal expenditure on science and technology
	Resources	Total consumption of water, soil and energy	Total water supply, built-up area, total gas supply and power supply of the whole society
Output	Expected outputs	Economic growth	Regional GDP (constant price in 2000)
		Social welfare	Average wages of employees and total retail sales of social consumer goods
		Pollution control capacity	Comprehensive utilization rate of industrial solid waste, centralized treatment rate of sewage treatment plant and harmless treatment rate of domestic garbage
	Unexpected outputs	Environmental pollution	Industrial wastewater discharge, industrial SO_2_ discharge and industrial soot discharge

#### 3.1.2. Explanatory variable: Vertical environmental regulation

In the existing literature, the quantification of VER is mainly carried out from two ideas: one is from the perspective of the frequency of specific texts (48). The will of the central government is often loaded in policy documents, and the higher the frequency, the more prominent the central government's determination to implement VER. However, the frequency of words in policy documents does not mean the actual strength of VER, so this paper adopts the second way of thinking, that is, starting with the “national control point” system. Since 2008, China has published a list of national key monitoring enterprises every year. The list is determined by the Ministry of Ecology and Environment in consultation with the provincial environmental protection departments and reported to the central office for assessment. All enterprises are directly supervised by the state on a regular basis, so it is more in line with the quantitative standard of VER. Shen et al. (30) and Zou et al. (19) have carried out research on the pollution reflux effect and river basin water pollution under VER based on the “national control point” system. With the rapid development of modern cities, automobile exhaust and residential heating coal have caused serious air pollution. Compared with waste gas, waste water pollution can more represent the pollution produced in industrial production. Based on this, this paper manually sort out the list of “national control points” wastewater enterprises, and matched the addresses of enterprises with each city to measure the actual strength of the central government's environmental regulation of each city.

#### 3.1.3. Moderating variable: Pollution dividend

How to quantify PD reasonably is a difficult problem. Taking Inner Mongolia as an object, Zhang (33) uses the gross value of industrial economy as the proxy variable of PD when studying whether PD caused pollution agglomeration, but this measurement method is too rough and has sample particularity. Later, Zhang and Yang expands PD into the difference between the actual treatment cost of waste gas and virtual treatment cost (49), but this ignores the benefits brought by pollution itself. In addition, these two measurement methods are from the perspective of enterprises, which is inconsistent with the perspective of local governments seeking PD proposed in this paper.

Based on the previous analysis, this paper divides PD into politically motivated pollution dividend (PPD) and economically motivated pollution dividend (EPD), in order to explain the motivation of local governments' passive implementation from different angles. PPD is naturally related to the political promotion of local officials, so we use the official tenure as a proxy variable. The reason is that maximizing visible achievements in a limited tenure is the first choice of local officials (50), which to some extent induces opportunistic pollution control behavior of local officials. The closer you get to the promotion node, the more obvious this tendency will be. EPD is related to local governments' competition for low-cost elements. In order to attract the inflow of low-cost factors and capital, local governments must gain priority in financial competition. Therefore, we refer to Guo and Jia (51) and Yu et al. (52), taking local government competition as a proxy variable, specifically the ratio of regional fiscal expenditure to fiscal revenue. The larger this indicator is, the higher the relative fiscal expenditure is, the better the investment environment in the jurisdiction is, and the more attractive it is to attract low-cost elements.

#### 3.1.4. Control variables

According to regional conditions and existing research (43, 53), we construct control variables from four aspects. ①Industrial structure (IS). Undoubtedly, a good level of industrial structure can reduce resource consumption and environmental pollution to the greatest extent. This paper uses Fu et al.'s (54) approach to measure IS by adopting the industrial structure advanced index; ②Foreign investment support (FIS). Generally speaking, the capital, technology and management experience brought by the influx of foreign investors will promote the regional green development in China, but there is also the phenomenon of “pollution refuge”. In this paper, the proportion of foreign investment in GDP is used to measure FIS. ③Ecological pressure (EP). The intensity of sewage discharge in densely populated areas is relatively high, but because of the larger groups affected by environmental pollution, more people are willing to participate in energy conservation and emission reduction activities (55). In this paper, the population density is adopted to measure EP. ④Education level (EDU). The improvement of education level may lead to the acceleration of industrial upgrading and technological innovation, but in the short term, the cost effect may not significantly improve GDE. In this paper, the proportion of the people studying in colleges and universities to the total population of the region is used to measure EDU.

### 3.2. Sample selection and data sources

In order to make up for the possible result bias caused by the macroscopic data of provincial level, this paper selects the data of city level to measure the spatial effect of VER. By eliminating some samples with discontinuous data and serious missing data, this paper finally forms a continuous data set of 267 cities. This paper selects the period from 2009 to 2018 for research. Objectively speaking, the national key monitoring enterprises that measure VER are only disclosed on the website of the Ministry of Ecology and Environment from 2009 to 2018. Limited by the availability of data, this paper only studies this decade. It is worth saying that what this paper discusses is not a problem with the particularity of time samples, but how the central and local governments can better coordinate governance under the special environmental decentralization system in China by using reasonable data. Therefore, the study of this period is also of long-term significance and will not affect the theoretical value of the conclusion of this paper.

The related data of VER comes from the Ministry of Ecology and Environment of China and is obtained by manual matching. The data of PPD comes from the resumes of officials on official website and Baidu Encyclopedia of various governments. After crawling and summarizing, it is matched with each city. When there is the phenomenon of “alternation of posts”, the officials who have served for a long time will be the local officials of that year. The related data of GDE, EPD, IS, FIS, EP and EDU come from the China Urban Statistical Yearbook, statistical yearbooks of various provinces and the National Bureau of Statistics. In view of the missing data in the sample, the interpolation method is used to complete it, and the related data expressed in monetary value is reduced by GDP index based on the year 2000. Descriptive statistics of each variable are shown in Table 2.

**Table 2:** Descriptive statistics.

**Variable name**	**Variable symbol**	**Variable explanation**	**Mean value**	**Standard deviation**
Green development efficiency	*GDE_*i, t*_*	See Table 1 (-) for specific input and output of GDE measured by super-efficiency SBM model	0.561	0.273
Vertical environmental regulation	*VER_*i, t*_*	Proportion of water pollution monitoring enterprises in national control points in cities	0.343	0.382
Pollution dividend	*PPD_*i, t*_*	Politically motivated pollution divdend: Term of office of local officials	2.167	1.476
	*EPD_*i, t*_*	Economically motivated pollution divdend: Fiscal expenditure/fiscal revenue	2.666	1.578
Industrial structure	*IS_*i, t*_*	Industrial structure upgrading index	6.491	0.368
Foreign investment support	*FIS_*i, t*_*	Actual utilization of foreign investment in GDP	1.427	3.286
Ecological pressure	*EP_*i, t*_*	Population density	4.520	3.366
Educational level	*EDU_*i, t*_*	The proportion of the number of ordinary colleges and universities to all the people in the region	10.605	0.629

### 3.3. Model construction

The spatial correlation between VER and GDE exists objectively, so a spatial econometric model with full consideration of spatial factors should be adopted when setting the model. Commonly used spatial models include spatial error model (SEM), spatial lag model (SLM) and spatial Durbin model (SDM), among which SDM has better performance in measuring the strategic interaction of neighboring individuals, so this paper adopts SDM for subject regression.

First of all, in order to test the nonlinear influence of VER on GDE, we introduce VER and its higher-order terms one after another, and set the model as follows:


(Model 1) $$\begin{array}{l} GDE_{i,t}=\rho W\times GDE_{i,t}+\alpha_{0}+\alpha_{1}VER_{i,t}+\lambda X_{i,t}+\theta_{1}W\times VER_{i,t}\\ \;+\gamma W\times X_{i,t}+\mu_{i}+\nu_{t}+\varepsilon_{i,t} \end{array}$$

(Model 2) $$\begin{array}{l} GDE_{i,t}=\rho W\times GDE_{i,t}+\alpha_{0}+\alpha_{1}VER_{i,t}+\alpha_{2}VER_{i,t}^{2}+\lambda X_{i,t}\\ \;+\theta_{1}W\times VER_{i,}+\theta_{2}W\times VER_{i,t}^{2}+\gamma W\times X_{i,t}+\mu_{i}+\nu_{t}\\ \;+\varepsilon_{i,t} \end{array}$$

(Model 3) $$\begin{array}{l} GDE_{i,t}=\rho W\times GDE_{i,t}+\alpha_{0}+\alpha_{1}VER_{i,t}+\alpha_{2}VER_{i,t}^{2}+\alpha_{3}VER_{i,t}^{3}+\lambda X_{i,t}\\ \;+\theta_{1}W\times VER_{i,}+\theta_{2}W\times VER_{i,t}^{2}+\theta_{2}W\times VER_{i,t}^{3}\\ \;+\gamma W\times X_{i,t}+\mu_{i}+\nu_{t}+\varepsilon_{i,t} \end{array}$$

Among them, *i* stands for prefecture-level city, and *t* stands for year. *W* is the spatial weight matrix, and the adjacency matrix is selected here. ρ is the spatial autoregressive coefficient, which indicates the influence of spatial aggregation among variables in different regions on the explained variables. *X*_*i, t*_ is a series of control variables, including *IS*_*i, t*_, *FIS*_*i, t*_, *EP*_*i, t*_, and *EDU*_*i, t*_; μ_*i*_ and ν_*t*_ represent regional effect and time effect, respectively; ε_*i, t*_ is a random disturbance term.

Secondly, in order to verify the behavior decision and strategic reflection of local governments under the central vertical regulation target, on the basis of Model 2, we further introduce the interaction between VER and PD to explore whether the local government's pursuit of PD affects the green governance effect of VER. The model is as follows:


(Model 4) $$\begin{array}{l} GDE_{i,t}=\rho W\times GDE_{i,t}+\alpha_{0}+\alpha_{1}VER_{i,t}+\alpha_{2}VER_{i,t}^{2}+\alpha_{3}PD_{i,t}\\ \;\times VER_{i,t}+\alpha_{4}PD_{i,t}\times VER_{i,t}^{2}+\alpha_{5}PD_{i,t}+\lambda X_{i,t}+\theta_{1}W\times VER_{i,t}\\ \;+\theta_{2}W\times VER_{i,t}^{2}+\theta_{3}W\times PD_{i,t}\times VER_{i,t}+\theta_{4}W\\ \;\times PD_{i,t}\times VER_{i,t}^{2}+\theta_{5}W\times PD_{i,t}+\gamma WX_{i,t}+\mu_{i}+\nu_{t}\\ \;+\varepsilon_{i,t} \end{array}$$

Among them, *PD*_*i, t*_ represents pollution dividend, and *PPD*_*i, t*_, *EPD*_*i, t*_ are calculated in turn. According to the moderating effect analysis paradigm with U-shaped curve as the main effect (56), if VER has a U-shaped influence on GDE, when the coefficients of *PD*_*i, t*_×*VER*${}_{i,t}^{2}$ are positive, it means that PD make the U-shaped curve steeper, which has a positive moderating effect; On the contrary, PD has a negative moderating effect.

## 4. Empirical results and analysis

### 4.1. Spatial autocorrelation test

In order to test the spatial correlation of the explained variables, this paper first calculates the Moran's index. As shown in Table 3, Moran's index of GDE is significantly not 0, and all of them have passed the 1% significance test except in 2009. Since 2012, Moran's index of GDE has been at a high level, and there is even a trend of increasing spatial agglomeration. Therefore, we think that GDE shows significant spatial dependence in geographical space, and it is suitable to use a spatial econometric model for analysis.

**Table 3:** Spatial autocorrelation test.

**Time**	**Moran's I**	**z**	***p*-value**
2009	0.037	0.969	0.166
2010	0.111	2.728	0.003
2011	0.122	3.267	0.001
2012	0.198	4.805	0.000
2013	0.185	4.500	0.000
2014	0.169	4.107	0.000
2015	0.142	3.463	0.000
2016	0.237	5.732	0.000
2017	0.232	5.604	0.000
2018	0.193	4.665	0.000

To improve the robustness of the results, we adopted a series of testing methods to determine the final spatial econometric model, as shown in Table 4. First of all, we further prove through LM test that spatial econometric regression is indeed better than general regression. After that, we use Hausman test to conclude that the fixed effect is more suitable than the random effect. In order to confirm the stability of SDM, we prove that SDM will not degenerate into SLM and SEM by LR and Wald test. Therefore, we prove that the SDM with fixed effect is the best choice.

**Table 4:** Spatial metrological inspection.

**Test**	**Model 1**	**Model 2**	**Model 3**
LM-lag	1,901.89^***^	1,889.89^***^	1,834.10^***^
Robust LM-lag	386.48^***^	420.53^***^	404.12^***^
LM-error	1,576.19^***^	1,528.63^***^	1,494.60^***^
Robust LM-error	60.74^***^	59.26^***^	64.62^***^
Hausman	28.82^***^	35.64^***^	40.89^***^
LR-SDM-SAR	32.89^***^	41.64^***^	44.94^***^
LR-SDM-SEM	37.05^***^	45.77^***^	49.38^***^
Wald-SDM-SAR	33.08^***^	41.96^***^	45.34^***^
Wald-SDM-SEM	23.36^***^	30.54^***^	34.62^***^

^***^Denote significance at 1% level.

### 4.2. Benchmark regression analysis

Table 5 reports the benchmark regression results of VER on GDE. The preliminary rules of explanatory variables are as follows: VER has a U-shaped influence on local GDE; In terms of spatial spillover, VER has an inverted N-shaped impact on GDE of neighboring areas.

**Table 5:** Spatial measurement results of VER on GDE.

**Variables**	**Model 1**	**Model 2**	**Model 3**
*VER_*i, t*_*	−0.161^***^ (−11.841)	−0.309^***^ (−11.867)	−0.353^***^ (−8.072)
*VER${}_{i,t}^{2}$*		0.0589^***^ (6.644)	0.109^***^ (2.845)
*VER${}_{i,t}^{3}$*			−0.010 (−1.375)
*FIS_*i, t*_*	−0.457^***^ (−5.258)	−0.451^***^ (−5.232)	−0.445^***^ (−5.162)
*IS_*i, t*_*	−0.124^***^ (−7.462)	−0.129^***^ (−7.862)	−0.133^***^ (−8.034)
*EDU_*i, t*_*	−0.072^**^ (−2.104)	−0.0737^**^ (−2.183)	−0.074^**^ (−2.198)
*EP_*i, t*_*	0.005^**^ (2.242)	0.007^***^ (2.888)	0.006^***^ (2.786)
*W* × *VER_*i, t*_*	0.078^***^ (3.157)	0.052 (1.141)	−0.057 (−0.745)
*W* × *VER${}_{i,t}^{2}$*		0.0185 (1.158)	0.150^**^ (2.085)
*W* × *VER${}_{i,t}^{3}$*			−0.028^*^ (−1.923)
*W*×*FIS_*i, t*_*	−0.467^***^ (−3.314)	−0.514^***^ (−3.670)	−0.503^***^ (−3.590)
*W* × *IS_*i, t*_*	0.004 (0.590)	0.00441 (0.652)	0.006 (0.875)
*W* × *EDU_*i, t*_*	0.041 (0.660)	0.0278 (0.455)	0.018 (0.289)
*W* × *EP_*i, t*_*	−0.012^***^ (−3.602)	−0.013^***^ (−3.736)	−0.012^***^ (−3.630)
ρ	0.191^***^ (7.689)	0.179^***^ (7.180)	0.176^***^ (7.011)
*sigma2_e*	0.062^***^ (36.419)	0.061^***^ (36.432)	0.061^***^ (36.438)
*Obs*	2,670	2,670	2,670

The t-statistics are given in parentheses; ^***^, ^**^, and ^*^ denote significance at 1, 5, and 10% level, respectively.

In order to accurately measure the marginal effect of green development of VER, we use partial differential method to decompose the results of Model 3 into three parts: direct effect, indirect effect and total effect, and make detailed analysis. The results are shown in Table 6.

**Table 6:** Decomposition results of the effect of VER on GDE.

**Variable**	**Direct effect**	**Indirect effect**	**Total effect**
*VER_*i, t*_*	−0.356^***^ (−7.980)	−0.139^*^ (−1.668)	−0.495^***^ (−5.380)
*VER${}_{i,t}^{2}$*	0.114^***^ (2.890)	0.200^**^ (2.458)	0.314^***^ (3.334)
*VER${}_{i,t}^{3}$*	−0.011 (−1.436)	−0.035^**^ (−2.113)	−0.046^**^ (−2.379)
*FIS_*i, t*_*	−0.469^***^ (−5.667)	−0.674^***^ (−4.463)	−1.143^***^ (−7.195)
*IS_*i, t*_*	−0.133^***^ (−8.386)	−0.019^*^ (−1.895)	−0.153^***^ (−7.368)
*EDU_*i, t*_*	−0.072^**^ (−2.122)	0.001 (0.021)	−0.071 (−0.810)
*EP_*i, t*_*	0.006^***^ (2.637)	−0.013^***^ (−3.548)	−0.007^**^ (−2.509)

The t-statistics are given in parentheses; ^***^, ^**^, and ^*^ denote significance at 1, 5, and 10% level, respectively.

From the direct effect, the coefficient of *VER*${}_{i,t}^{2}$ is significantly positive (α = 0.114, *p* < 0.01), that is, the impact of VER on the local GDE presents a U-shaped feature, which is consistent with the research conclusions of most scholars (57, 58). According to the calculation, when the intensity of VER exceeds 1.561, its green governance effect begins to appear. According to the current regulation level, only 1.49% of the samples fall to the right of the inflection point. As a result, under the heavy pressure of the central government and the lag of information, the productivity of enterprises has been negatively affected, whether they are upgraded locally or transferred nearby. In the short term, the green governance effect of VER has not been highlighted, and most cities are still in a strange circle of “weak regulation”. In the long run, VER has a positive green governance effect, and polluting enterprises will pay more attention to the pressure of emission reduction from the central government and gradually construct the management strategy of coordinated development of economic growth and environmental protection. So far, the hypothesis 1 has been proved.

From the indirect effect, VER have an inverted N-shaped impact on GDE of neighboring areas. According to the calculation, when VER is in the range of (0, 0.138), it has a negative spillover effect on GDE of neighboring areas, that is, there is the phenomenon that early polluting enterprises fled to the areas with loose regulations, resulting in the surrounding areas becoming “pollution shelters”. When VER is in the range of (0.138, 3.012), it has a positive spillover effect on GDE of neighboring areas. This is called the scale effect of environmental regulation: when polluting enterprises can't profit from the regulation difference, they will have to invest some funds in innovative technology research and development to promote the upgrading of green production structure, thus driving the “emission reduction” and “quality improvement” in the surrounding areas in parallel. When the intensity of VER is >3.012, it has a negative spillover effect on GDE of neighboring areas. It is because the scale effect of VER has a certain range: excessive VER pressure can not stimulate enterprises to generate transformation power, but makes neighboring areas become victims of pollution transfer again. At present, the proportion of samples in the positive spillover range is 73.8% of the total samples, which shows that although VER is not effective in the local area, it can well promote the green development of the surrounding areas. So far, hypothesis 2 has been proved. In particular, the spillover effect of VER on GDE has changed from negative to positive.

In addition, the control variables also bring some information. FIS and IS can significantly inhibit the GDE of local and neighboring areas, which shows that due to the need of local government to develop economy, green development is not the first goal in setting the entry threshold of foreign capital, and the imbalance of industrial structure is also one of the reasons for the lack of green “engine” power. The influence of EDU is only significant in the local area, and it has a slight inhibitory effect on GDE. This may be because abundant educational resources attract talents to gather and economic activities increase correspondingly, and the resulting pressure of pollution control leads to the slowdown of green development. EP can promote GDE, because when the number of people affected by the negative effects of environmental pollution increases, the spontaneous actions to promote green development will also increase, which is in line with expectations; As for the negative spillover effect of EP, this may be because when the population gathers in the local area, the number of people affected by pollution in neighboring areas will decrease, thus voluntary environmental protection behavior will also decrease, resulting in the absence of public power in the process of green development.

### 4.3. Analysis of moderating effect

Because China's environmental governance is dominated by the “centralization-decentralization” paradigm, the “self-interest” motivation of local governments may affect the green governance effect of VER. Based on Model 4, this paper explores the moderating effects of PPD and EPD on the relationship between VER and GDE, and the results are shown in Table 7.

**Table 7:** Test results of moderating effect of PD on green governance effect of VER.

**Variable**	**Model 4:** ***PD_*i, t*_ = PPD_*i, t*_***	**Model 4:** ***PD_*i, t*_ = EPD_*i, t*_***
*VER_*i, t*_*	−0.363^***^ (−7.923)	−0.212^***^ (−4.563)
*VER${}_{i,t}^{2}$*	0.090^***^ (5.362)	−0.005 (−0.225)
*PD_*i, t*_*	−0.007 (−1.188)	0.0396^***^ (7.48)
*PD_*i, t*_*×*VER_*i, t*_*	0.024 (1.422)	−0.054^***^ (−2.616)
*PD_*i, t*_*×*VER${}_{i,t}^{2}$*	−0.014^**^ (−2.050)	0.051^***^ (2.656)
*W* × *VER_*i, t*_*	−0.038 (−0.438)	0.039 (0.449)
*W* × *VER${}_{i,t}^{2}$*	0.025 (0.774)	0.017 (0.367)
*W* × *PD_*i, t*_*	0.012 (1.071)	0.001 (0.064)
*W* × *PD_*i, t*_*×*VER_*i, t*_*	0.030 (0.954)	0.001 (0.011)
*W* × *PD_*i, t*_*×*VER${}_{i,t}^{2}$*	−0.001 (−0.048)	0.006 (0.154)
*Additional Controls*	Yes	Yes
ρ	0.174^***^ (6.936)	0.159^***^ (6.243)
*sigma2_e*	0.060^***^ (36.436)	0.0591^***^ (36.446)
Obs	2,670	2,670

The t-statistics are given in parentheses; ^***^ and ^**^ denote significance at 1% and 5% level.

It can be seen from Table 7 that the coefficients of *PPD*_*i, t*_× *VER*${}_{i,t}^{2}$ are significantly negative (α = −0.014, *p* < 0.05), that is, PPD will ease the original U-shaped curve of VER, which has a negative moderating effect. Under the current background of an uncoordinated promotion incentive mechanism, although the central government tries its best to break the inertia assessment thinking of economic catch-up, the political promotion intention of local governments has not been completely transformed into the endogenous driving force of green development. The limited resources in the jurisdiction make it impossible for local governments to take into account the dual goals of economic development and environmental protection. In order to enter the fast lane of promotion, local governments will acquiesce or even help some polluting enterprises to whitewash their pollution discharge behavior, which will help these enterprises to get rid of the problem of productivity decline caused by the “cost compliance effect”, and to some extent weaken the restraining effect of VER on GDE in the short term. In the long run, environmental protection achievements have not been regarded as a necessity for promotion in the official promotion assessment system. Constrained by limited resources, local officials will not go to great lengths to urge polluting enterprises to carry out green transformation. Although the impact of VER on GDE has turned from negative to positive, its due effectiveness is still affected by the negative adjustment of PPD. In terms of spatial spillover, this paper has not found any evidence that the PPD moderates the effect of VER in neighboring areas. So far, the hypothesis 3 has been partially established, and the local government's pursuit of PPD should be taken seriously by the central policymakers.

The coefficients of *EPD*_*i, t*_× *VER*${}_{i,t}^{2}$ are significantly positive (α = 0.051, *p* < 0.01), that is, EPD aggravates the original U-shaped curve of VER, which has a positive moderating effect. Before the turning point, local governments tried to attract liquid resources to achieve economic growth, but the quality levels of the rapidly incurred elements were uneven, and the pressure on pollution control also increased. Although polluting enterprises are under the regulatory pressure of the central government, a large number of low-quality production factors can not change their high-pollution and high-return business strategies in a short period, but have caused overcrowding in economic activities, which makes the road to green development more difficult. In a word, the local government's short-term pursuit of EPD makes VER more obvious in inhibiting GDE. After the inflection point, EPD highlights the green governance effect of VER. This is because the gathering of high-quality elements such as technology and talents can promote the cleaner development of enterprise technology after the region completes industrial upgrading to adapt to green development. The coordination of economic and environmental protection goals has been greatly improved under the guidance of green technology. In terms of spatial spillovers, the moderating effect of EPD is not significant. So far, the hypothesis 4 has been partially established, that is, in the long run, the local government's pursuit of EPD is conducive to injecting vitality into the green economy.

### 4.4. Robustness test

In order to test the robustness of the above results, based on model (3) and model (4), this paper carries out robustness tests from three aspects: changing the spatial weight matrix, adding control variables and changing the spatial model. The results are shown in Tables 8, 9.

**Table 8:** The robustness test of effect of VER on GDE.

**Variables**	**Change weight matrix** **(1)**	**Add control** **variables** **(2)**	**Replace the spatial model** **(3)**	**Replace the explained variable** **(4)**
*VER_*i, t*_*	−0.330^***^ (−7.734)	−0.347^***^ (−7.879)	−0.344^***^ (−8.007)	−0.270^***^ (−6.297)
*VER${}_{i,t}^{2}$*	0.103^***^ (2.704)	0.106^***^ (2.759)	0.107^***^ (2.825)	0.202^***^ (5.382)
*VER${}_{i,t}^{3}$*	−0.010 (−1.405)	−0.010 (−1.325)	−0.010 (−1.340)	−0.034 (−1.405)
*W* × *VER_*i, t*_*	−2.027^***^ (−3.787)	−0.050 (−0.635)	——	0.161^**^ (2.174)
*W* × *VER${}_{i,t}^{2}$*	2.690^***^ (4.755)	0.142^*^ (1.919)	——	0.003 (0.038)
*W* × *VER${}_{i,t}^{3}$*	−0.517^***^ (−4.421)	−0.026^*^ (−1.750)	——	−0.014 (−0.972)
*Additional Controls*	Yes	Yes	Yes	Yes
ρ	0.617^***^ (7.013)	0.181^***^ (7.117)	lambda 0.201^***^ (8.049)	0.283^***^ (11.834)
*sigma2_e*	0.056^***^ (36.391)	0.061^***^ (36.431)	0.062^***^ (36.415)	0.058^***^ (36.294)
*Obs*	2,670	2,670	2,670	2,670

The t-statistics are given in parentheses; ^***^, ^**^, and ^*^ denote significance at 1, 5, and 10% level, respectively.

**Table 9:** The robustness test of the moderating effect of PD on the green governance effect of VER.

**Variable**	**Change weight matrix**		**Add control variables**		**Replace the spatial model**		**Replace the explained variable**	
	***PD**_*i, t*_ = **PPD**_*i, t*_* **(1)**	***PD**_*i, t*_ = **EPD**_*i, t*_* **(2)**	***PD**_*i, t*_ = **PPD**_*i, t*_* **(3)**	***PD**_*i, t*_ = **EPD**_*i, t*_* **(4)**	***PD**_*i, t*_ = **PPD**_*i, t*_* **(5)**	***PD**_*i, t*_ = **EPD**_*i, t*_* **(6)**	***PD**_*i, t*_ = **PPD**_*i, t*_* **(7)**	***PD**_*i, t*_ = **EPD**_*i, t*_* **(8)**
*VER_*i, t*_*	−0.339^***^ (−7.496)	−0.196^***^ (−4.284)	−0.387^***^ (−8.957)	−0.227^***^ (−4.874)	−0.361^***^ (−7.853)	−0.204^***^ (−4.511)	−0.246^***^ (−5.808)	−0.171^***^ (−3.770)
*VER${}_{i,t}^{2}$*	0.088^***^ (5.291)	−0.010 (−0.475)	0.098^***^ (6.051)	−0.007 (−0.326)	0.091^***^ (5.394)	0.006 (−0.267)	0.063^***^ (3.973)	0.142^***^ (6.736)
*PD_*i, t*_*	−0.007 (−1.112)	0.034^***^ (6.536)	−0.011^*^ (−1.888)	0.049^***^ (8.635)	−0.008 (−1.255)	0.043^***^ (8.672)		0.043^***^ (8.275)
*PD_*i, t*_*×*VER_*i, t*_*	0.022 (1.330)	−0.057^***^ (−2.775)	0.038^**^ (2.361)	−0.050^**^ (−2.419)	0.029^*^ (1.773)	−0.051^**^ (−2.515)	0.058^***^ (3.657)	−0.184^***^ (−9.146)
*PD_*i, t*_*×*VER${}_{i,t}^{2}$*	−0.015^**^ (−2.204)	0.054^***^ (2.797)	−0.019^***^ (−2.844)	0.054^***^ (2.846)	−0.016^**^ (−2.359)	0.051^**^ (2.693)	−0.014^**^ (−2.078)	0.141^***^ (7.633)
*W* × *VER_*i, t*_*	−0.382 (−0.678)	1.124^*^ (1.824)	−0.003 (−0.031)	−0.008 (−0.092)	——	——	0.095 (1.195)	0.353^***^ (4.230)
*W* × *VER${}_{i,t}^{2}$*	0.165 (0.767)	−0.891^**^ (−2.328)	0.014 (0.449)	0.025 (0.546)	——	——	−0.035 (−1.120)	−0.022 (−0.494)
*W* × *PD_*i, t*_*	0.073 (1.107)	0.174^***^ (3.177)	0.012 (1.091)	0.003 (0.275)	——	——	0.006 (0.556)	0.012 (1.469)
*W* × *PD_*i, t*_*×*VER_*i, t*_*	0.036 (0.185)	−0.962^***^ (−2.851)	0.017 (0.552)	0.007 (0.166)	——	——	0.030 (0.960)	−0.017 (−0.404)
*W* × *PD_*i*_,*×*VER${}_{i,t}^{2}$*	0.055 (0.638)	1.043^***^ (3.188)	0.004 (0.288)	0.008 (0.211)	——	——	−0.004 (−0.344)	−0.065^*^ (−1.648)
*Additional Controls*	Yes	Yes	Yes	Yes	Yes	Yes	Yes	Yes
ρ	0.647^***^ (7.727)	0.562^***^ (5.879)	0.181^***^ (7.124)	0.161^***^ (6.231)	lambda 0.204^***^ (8.156)	lambda 0.177^***^ (7.042)	0.277^***^ (11.531)	0.275^***^ (11.454)
*sigma2_e*	0.0560^***^ (36.369)	0.058^***^ (36.394)	0.060^***^ (36.430)	0.058^***^ (36.447)	0.062^***^ (36.402)	0.060^***^ (36.438)	0.058^***^ (36.302)	0.056^***^ (36.294)
Obs	2,670	2,670	2,670	2,670	2,670	2,670	2,670	2,670

The t-statistics are given in parentheses;^***^, ^**^, and ^*^ denote significance at 1, 5, and 10% level, respectively.

First, change the spatial weight matrix. Because the adjacency matrix may weaken the relationship between similar but non-adjacent samples, this paper uses the inverse distance matrix constructed by latitude and longitude to describe the spatial weight. According to the results in column (1) of Table 8, columns (1) and (2) of Table 9, combined with Tables 6, 7, it can be seen that the main conclusions drawn above are basically confirmed to be robust, except for slight changes in coefficients. In addition, we also find new evidence: under the inverse distance matrix, EPD has a negative moderating effect on spatial spillover (θ = 1.043, *p* < 0.01), that is, when VER is in the positive spillover range, the local government's pursuit of EPD will inhibit the regional green win-win situation; When VER is in the negative spillover range, the local government's pursuit of EPD will inhibit the situation that “one party benefits while the other pays”. This is mainly due to the fact that when polluting enterprises scramble to flee, local governments will lose part of their economic support, so they will increase preferential policies to attract enterprises to stay, thus inhibiting the spatial spillover effect of VER.

Second, add control variables. Regression has included four control variables: IS, FIS, EDU and EP. In order to avoid being affected by missing variables, this paper add GDP per capita, the degree of government intervention and the level of financial development into the equation to test the robustness of the existing results. According to the relevant tables, it can be seen that the coefficient and significance of the main explanatory variables can basically prove that the benchmark research results are relatively stable and not affected by the missing variables. Among them, we find that the significance of spatial spillover effect of VER has decreased, but its positive spillover interval has been prolonged, which shows that the triple consideration of economic development, government intervention and financial development level will optimize the spatial spillover effect of VER, which is conducive to the common green development in the region.

Third, replace the space model. Anselin points out that there are two ways to introduce spatial correlation into ordinary econometric models: spatially weighted explained variables and spatially weighted error terms (59). In order to test the robustness of the previous regression results, the spatial correlation is measured by the spatial weighted error term, that is, the spatial error model is used for regression. According to the relevant tables, it can be seen that the coefficients and significance of the main explanatory variables have not changed significantly, indicating that the benchmark regression results have good robustness. In addition, the lambda coefficient further proves that GDE among cities shows strong spatial dependence, and geographical proximity is conducive to the formation of green clusters.

Fourth, replace the explained variable. According to the method of Kou and Han (48), this paper recalculates the GDE, that is, only considering the expected output of economic growth and adopting the SBM model with unexpected output. The correlation results can prove the robustness of benchmark regression. It is worth mentioning that, as shown in column (4) of Table 8, it can be seen that VER and local GDE still show a stable U-shaped curve, but the inflection point obviously comes earlier; In terms of spatial spillover, VER can significantly promote GDE in surrounding cities. This means that if only one-dimensional expected output is considered, the green governance effect of VER will overcome the growth loss caused by cost compliance more quickly.

## 5. Further discussion

### 5.1. Time heterogeneity analysis

There is a widespread problem of regional division in China's environmental governance, which makes it easier for local governments to profit from the negative externalities of pollution and make strategic policies. Therefore, strengthening cross-regional environmental governance has become an important starting point to restrain the self-interest behavior of local governments and accelerate the green development process. In 2012, the 18th National Congress of the Communist Party of China was successfully held, with “green development”, “ecological civilization” and “beautiful China” as keywords, demonstrating China's determination to win the tough battle of pollution prevention and control. In order to improve the efficiency of environmental governance and solve the “beggar-thy-neighbor” problem, since 2012, cross-regional cooperative governance (CCG) has also been widely used and discussed, and regional integration “going green” has gradually become the policy direction. Thus, this paper takes 2012 as the split time point to explore whether there is time heterogeneity in benchmark regression results.

Table 10 shows the decomposition results of the effect of VER on GDE in different time periods. From the direct effect point of view, the coefficient of *VER*${}_{i,t}^{2}$ is significantly positive. In the period of 2009–2012, the coefficient is 0.143, which passes the significance test of 5%. After 2012, the coefficient is 0.088, which passes the significance test of 1%. This shows that the U-shaped relationship between VER and GDE is stable, but after 2012, the U-shaped relationship becomes slower and the inflection point moves to the right. The slowing down of the U-shaped relationship indicates that the CCG can alleviate the early inhibitory effect of VER on GDE. This is because the problem of fragmentation governance has been improved, and some repeated costs in the process of green governance have been avoided, so the productivity of enterprises can be improved and the negative effect of VER has been alleviated. The turning point to the right shows that the CCG has delayed the emergence of the green governance effect of VER. This is because there is still the problem of uneven coordination of the interests of various subjects in CCG, and some regions still need to sacrifice individual interests to obtain collective interests, so the green governance effect appears at a later time. From the indirect effect, VER has no negative spillover effect on GDE of neighboring areas in two time periods, which shows that CCG has restrained the pollution transfer phenomenon caused by VER to some extent, and is conducive to the advancement of regional green governance.

**Table 10:** Decomposition results of effects of VER on GDE in different time periods.

**Variable**	**2009–2012**			**2013–2018**		
	**Direct effect**	**Indirect effect**	**Total effect**	**Direct effect**	**Indirect effect**	**Total effect**
*VER_*i, t*_*	−0.361^***^ (−4.802)	−0.189 (−1.308)	−0.550^***^ (−3.521)	−0.331^***^ (−6.029)	−0.075 (−0.740)	−0.405^***^ (−3.568)
*VER${}_{i,t}^{2}$*	0.143^**^ (2.112)	0.181 (1.251)	0.324^**^ (1.965)	0.088^*^ (1.831)	0.155 (1.569)	0.243^**^ (2.114)
*VER${}_{i,t}^{3}$*	−0.019 (−1.384)	−0.0310 (−1.005)	−0.050 (−1.405)	−0.005 (−0.567)	−0.028 (−1.379)	−0.033 (−1.401)

The t-statistics are given in parentheses;^***^, ^**^, and ^*^ denote significance at 1, 5, and 10% level, respectively.

Table 11 reports the test results of the moderating effect of PD on the green governance effect of VER in different time periods. First of all, the negative moderating effect of PPD on U-shaped curve became significant after 2012 (α = −0.015, *p* < 0.1), which shows that the CCG can't prevent the local government from obstructing the local green process due to promotion. This is because the zero-sum game of political promotion of local officials greatly squeezes the cooperation space with the goal of environmental coordination. Under the constraint of limited resources, local officials usually don't “go to great lengths” in cooperative governance. In terms of spatial spillover, we find that after 2012, PPD positively moderates the negative spillover of VER (θ = 0.112, *p* < 0.01), indicating that the CCG effectively inhibites the “beggar-my-neighbor” behavior caused by political promotion. This is because CCG has strengthened the connection between governments at the same level, and opportunistic behaviors such as pollution discharge at the border have been restrained to some extent.

**Table 11:** Test results of moderating effect of PD on green governance effect of VER in different time periods.

**Variable**	**2009–2012**		**2013–2018**	
	***PD_*i, t*_ = PPD_*i, t*_***	***PD_*i, t*_ = EPD_*i, t*_***	***PD_*i, t*_ = PPD_*i, t*_***	***PD_*i, t*_ = EPD_*i, t*_***
*VER_*i, t*_*	−0.332^***^ (−3.753)	−0.088 (−1.122)	−0.353^***^ (−6.122)	−0.247^***^ (−4.302)
*VER${}_{i,t}^{2}$*	0.078^*^ (1.777)	−0.001 (−0.040)	0.091^***^ (4.766)	0.011^*^ (0.419)
*PD_*i, t*_*	−0.005 (−0.536)	0.027^***^ (3.319)	−0.005 (−0.673)	0.048^***^ (7.046)
*PD_*i, t*_*×*VER_*i, t*_*	0.022 (0.768)	−0.074^**^ (−2.277)	0.021 (0.925)	−0.029 (−1.119)
*PD_*i, t*_*×*VER${}_{i,t}^{2}$*	−0.010 (−0.665)	0.023 (0.727)	−0.015^*^ (−1.738)	0.039^*^ (1.664)
*W* × *VER_*i, t*_*	0.167 (1.051)	−0.420^***^ (−2.861)	−0.182^*^ (−1.658)	0.209^**^ (1.986)
*W* × *VER${}_{i,t}^{2}$*	0.002 (0.022)	0.0341 (0.454)	0.058 (1.546)	0.029 (0.498)
*W* × *PD_*i, t*_*	0.049^***^ (2.705)	−0.017 (−1.162)	−0.008 (−0.525)	0.018^*^ (1.696)
*W* × *PD_*i, t*_*×*VER_*i, t*_*	−0.085 (−1.578)	0.106 (1.471)	0.112^***^ (2.627)	−0.028 (−0.545)
*W* × *PD_*i, t*_*×*VER${}_{i,t}^{2}$*	0.016 (0.539)	0.076 (1.121)	−0.020 (−1.224)	−0.038 (−0.777)
*Additional Controls*	Yes	Yes	Yes	Yes
*rho*	0.199^***^ (4.860)	0.190^***^ (4.614)	0.165^***^ (5.200)	0.134^***^ (4.112)
*sigma2_e*	0.059^***^ (23.023)	0.0580^***^ (23.029)	0.0580^***^ (28.238)	0.0556^***^ (28.253)
Obs	1068	1068	1602	1602

The t-statistics are given in parentheses;^***^, ^**^, and ^*^ denote significance at 1, 5, and 10% level, respectively.

Secondly, the positive moderating effect of EPD on U-shaped curve has become significant since 2012 (α = 0.039, *p* < 0.1), which indicates that CCG can effectively promote the scale effect of factor agglomeration. This is because the CCG is conducive to the sharing of high-quality resources among local governments and optimizes the green governance effect of VER. In terms of spatial spillover, the moderating effect of EPD is not significant in the two periods, which is consistent with the benchmark regression results.

### 5.2. Analysis of regional heterogeneity

The Yangtze River Economic Zone (YREZ) and the Yellow River Eco-economic Belt (YREEB), as pioneers of China's green development, are the focus of environmental regulation. YREZ relies on the golden waterway, and the highly polluting industries are developing rapidly; YREEB is dominated by agriculture, and the use of a large number of pesticides and fertilizers will also bring water pollution. Although the construction of economic belt benefits from the economies of scale generated by the concentration of factors, the over-crowding of economic activities may cause the pollution superposition effect, making the regional pollution more serious and affecting the green governance effect of VER. Based on this, we take YREZ and YREEB as the objects for grouping regression, so as to explore whether there is regional heterogeneity in the regression results.

Table 12 shows the decomposition results of the effect of VER on GDE in the two economic belts. From the direct effect point of view, VER and GDE in the two economic belts have a U-shaped relationship. The U-shaped curve of the YREEB is steeper (α = 1.058, *p* < 0.1), which indicates that the long-term green governance effect of VER is better in the YREEB. This may be due to the relatively small economic volume and pollution control pressure of YREEB, which has great green development potential. The turning point of the U-shaped curve in YREZ is earlier, which indicates that VER has entered the stage of “innovation compensation” earlier in YREZ. This is mainly because the high level of technological innovation in YREZ, so polluting enterprises can quickly overcome the negative impact brought by the earlier stage of “cost compliance”. From the indirect effect, we find that VER has a positive spatial spillover effect of green governance in YREZ (θ = 0.215, *p* < 0.1), while the spatial spillover effect in YREEB is not significant, which indicates that VER has become an important thrust for the green integrated development of the YREZ.

**Table 12:** Decomposition results of effects of VER on GDE in two economic belts.

**Variable**	**YREZ**			**YREEB**		
	**Direct effect**	**Indirect effect**	**Total effect**	**Direct effect**	**Indirect effect**	**Total effect**
*VER_*i, t*_*	−0.373^***^ (−5.704)	0.215^*^ (1.882)	−0.158 (−1.216)	−0.764^***^ (−3.036)	0.107 (0.181)	−0.657 (−0.931)
*VER${}_{i,t}^{2}$*	0.095^*^ (1.883)	0.031 (0.322)	0.126 (1.095)	1.058^*^ (1.669)	0.086 (0.059)	1.145 (0.655)
*VER${}_{i,t}^{3}$*	−0.005 (−0.528)	−0.012 (−0.632)	−0.017 (−0.744)	−0.565 (−1.297)	−0.141 (−0.140)	−0.705 (−0.589)

The t-statistics are given in parentheses; ^***^ and ^*^ denote significance at 1% and 10%.

Table 13 reports the test results of the moderating effect of PD on the green governance effect of VER in two economic belts. First of all, PPD plays a negative role in moderating the local green governance effect of VER in the YREZ (α = −0.024, *p* < 0.01), but we have not found such evidence in YREEB. This is because that YREZ, as an important strategic area in China, has multi-dimensional strategic objectives, such as innovation-driven industrial transformation and upgrading, actively promoting new urbanization, and striving to build a new pattern of opening up. Officials in office will not regard green development as the only achievement construction goal, so it will weaken the green governance effect of VER. In terms of spatial spillover, the moderating effect of PPD is not significant in the two economic belts.

**Table 13:** The results of the moderating effect of PD on the green governance effect of VER in the two economic belts.

**Variable**	**YREZ**		**YREEB**	
	***PD**_*i, t*_ = **PPD**_*i, t*_*	***PD**_*i, t*_ = **EPD**_*i, t*_*	***PD**_*i, t*_ = **PPD**_*i, t*_*	***PD**_*i, t*_ = **EPD**_*i, t*_*
*VER_*i, t*_*	−0.496^***^ (−7.460)	−0.179^**^ (−2.540)	−0.705^***^ (−3.334)	0.148 (0.689)
*VER${}_{i,t}^{2}$*	0.123^***^ (5.934)	0.017 (0.612)	0.396 (1.634)	−0.359 (−1.495)
*PD_*i, t*_*	−0.019^**^ (−2.012)	0.046^***^ (5.839)	−0.019 (−1.375)	0.029^***^ (3.322)
*PD_*i, t*_*×*VER_*i, t*_*	0.063^***^ (2.712)	−0.072^**^ (−2.264)	0.098 (1.312)	−0.201^***^ (−3.248)
*PD_*i, t*_*×*VER${}_{i,t}^{2}$*	−0.024^***^ (−2.989)	0.027 (1.033)	−0.066 (−0.772)	0.210^**^ (2.527)
*W* × *VER_*i, t*_*	0.264^**^ (2.149)	−0.008 (−0.062)	−0.038 (−0.089)	0.175 (0.421)
*W* × *VER${}_{i,t}^{2}$*	−0.037 (−0.940)	−0.013 (−0.219)	0.351 (0.760)	−0.057 (−0.125)
*W* × *PD_*i, t*_*	0.012 (0.691)	−0.012 (−0.941)	0.020 (0.683)	0.009 (0.526)
*W* × *PD_*i, t*_*×*VER_*i, t*_*	0.000 (0.005)	0.105 (1.615)	0.137 (0.932)	0.019 (0.140)
*W* × *PD_*i, t*_*×*VER${}_{i,t}^{2}$*	0.004 (0.271)	0.028 (0.509)	−0.218 (−1.390)	−0.033 (−0.183)
*Additional Controls*	Yes	Yes	Yes	Yes
*rho*	0.136^***^ (3.491)	0.094^**^ (2.291)	0.267^***^ (6.050)	0.264^***^ (5.916)
*sigma2_e*	0.050^***^ (22.976)	0.057^***^ (22.982)	0.057^***^ (20.349)	0.057^***^ (20.344)
Obs	1,060	1,060	840	840

The t-statistics are given in parentheses; ^***^ and ^**^ denote significance at 1% and 5%.

Secondly, there is a significant difference in the moderating effect of EPD on the local green governance effect of VER. In YREZ, EPD has a linear negative moderating effect (α = −0.072, *p* < 0.05), that is, the local government's means of attracting the concentration of elements will weaken the green governance effect of VER. This is because YREZ is already an area where the elements are concentrated, and too many elements can easily lead to inefficient governance. In YREEB, EPD positively moderates the U-shaped governance curve of VER (α = 0.210, *p* < 0.05). This is because, compared with YREZ, the development of YREEB is largely dependent on the development of resource endowments, so the gathering of high-quality elements will greatly promote the green transformation of its industry and increase its green development vitality. In terms of spatial spillovers, the moderating effect of EPD is not significant in the two economic belts.

## 6. Conclusion and prospect

Based on the governance system of China's central-local decentralization system, this paper mainly studies the spatial impact of VER on GDE by using SDM, and adds the PPD and EPD as the moderating variables to explore whether the local government's pursuit of PD affects the green governance effect of VER. The main conclusions of this paper are as follows:

(1) VER has a U-shaped impact on the local GDE. Although it is a sharp weapon in the process of green development in the long run, most cities have not passed the inflection point under the existing VER level. VER has an inverted N-shaped impact on GDE of neighboring areas, and most cities are in a positive spillover range, which shows that VER has great potential to drive the green development of the region.
(2) PPD has a negative moderating effect on the local green governance effect of VER, which makes VER lose its due green governance effect in the long run; EPD has a positive moderating effect, which can drive industrial upgrading through factor agglomeration to strengthen the driving force of VER to drive green development. In terms of spatial spillovers, neither of the two types of PD has a significant moderating effect.
(3) In terms of time heterogeneity, CCG started in 2012 leads to the slow effect of VER, but it also inhibited the short-term inhibition of VER and its pollution transfer phenomenon. From the perspective of PPD, CCG can hardly prevent local governments from obstructing the local green development process by pursuing political promotion, but it can effectively restrain the phenomenon of “beggar-thy-neighbor” caused by political promotion; From the perspective of EPD, CCG can promote the gathering of high-quality elements and optimize the long-term governance effect of VER, but has no significant impact on spatial spillover.
(4) In terms of regional heterogeneity, the local green governance effect of VER is better in the YREEB, and its neighboring green governance effect is better in the YREZ. From the perspective of PPD, in YREZ, its local green governance effect on VER has a negative moderating effect, which is not significant in YREEB. From the perspective of EPD, it has a negative linear moderating effect on the local green governance effect of VER in the YREZ, but it has a positive moderating effect in YREEB. In addition, in terms of spatial spillovers, the moderating effect of PPD and EPD is not significant in the two economic belts.

Combined with the research conclusion, in order to narrow the EPI index gap between China and Denmark and improve China's green development level, we put forward suggestions from the following three aspects:

(1) Establish a central-local collaborative governance mechanism from the perspective of decentralization. In view of the long-term outstanding green governance effect of VER, the power of environmental regulation should be promoted to move up, so as to avoid the dilemma of environmental governance caused by excessive political space for local governments. When the central government implements VER, it should fully consider the cost of industrial upgrading and pollution transfer, provide certain funds to guide the regional “green” transformation, and at the same time, strengthen the environmental supervision of the central government to local governments, such as setting up supervision institutions at all levels to avoid the information asymmetry between the central government and the local government, and promote China's green governance process through the concerted efforts of the central government and the local government.
(2) Design the performance evaluation standard of environmental protection incentive type. In view of the negative moderating effect of PPD on the effect of VER, we should comprehensively assess the construction of ecology and people's livelihood, so as to avoid officials seeking PD for promotion. Although the “one-vote” system of environmental protection has improved officials' management of environmental protection indicators, the singleness of emission reduction indicators can't promote the formation of a long-term environmental governance mechanism. Therefore, the assessment indicators should be further involved from the aspects of environmental governance efficiency and public evaluation results to strengthen the construction of local green achievements.
(3) Improve the system of CCG. CCG can reduce the negative spillover of environmental governance through central leadership, horizontal coordination and multiple driving. First, avoid the negative overflow caused by the loopholes of laws and regulations drilled by enterprises at a lower cost, resulting in the crisis of institutional legitimacy and the crisis of “environmental-social” governance. Second, weaken the local government's “free-riding” mentality, and promote the environmental regulation to conform to the matching degree between the current economic situation and the industrial layout, and at the same time have a positive impact on the local and surrounding residents' wellbeing. At the same time, an effective feedback platform of environmental network can be established to help residents express their wishes through the network and weaken the “free-riding” effect, so that environmental regulation can have a positive effect on local and neighboring residents' wellbeing while conforming to the current situation of economic development and matching the industrial layout.
(4) Create the green growth pole position of the two major economic belts. The YREZ should raise the investment threshold reasonably to prevent the pollution control from becoming more difficult due to excessive factors gathering. At the same time, the YREZ should adhere to the road of green development driven by innovation, ensure that VER passes the cost-following stage as soon as possible, and promote the transformation and upgrading of polluting enterprises. The YREEB should combine factor endowments, develop characteristic eco-industries and avoid the old road of “pollution first, then treatment.” At the same time, it is necessary to further strengthen regional linkage and expand the overall opening-up level of the YREEB with the help of the environmental regulation policy promulgated by the central government to attract the inflow of funds and talents.

## Data availability statement

The datasets presented in this article are not readily available because they were collected by hand from several public platforms in China. Requests to access the datasets should be directed to SD, 210413120004@hhu.edu.cn.

## Author contributions

SD and HP: conceptualization. SD and ZM: data curation and methodology. SD and NW: formal analysis and investigation. ZM and HP: supervision. SD, ZM, and JR: writing–review and validation. All authors contributed to the article and approved the submitted version.
